# From Then to Now and Beyond: Exploring How Machine Learning Shapes Process Design Problems

**DOI:** 10.69997/sct.116002

**Published:** 2024-07-10

**Authors:** Burcu Beykal

**Affiliations:** a Department of Chemical & Biomolecular Engineering, University of Connecticut, Storrs, CT, USA; b Center for Clean Energy Engineering, University of Connecticut, Storrs, CT, USA

**Keywords:** Surrogate modeling, Artificial Intelligence, Historical view, Data-driven analysis, Process synthesis

## Abstract

Following the discovery of the least squares method in 1805 by Legendre and later in 1809 by Gauss, surrogate modeling and machine learning have come a long way. From identifying patterns and trends in process data to predictive modeling, optimization, fault detection, reaction network discovery, and process operations, machine learning became an integral part of all aspects of process design and process systems engineering. This is enabled, at the same time necessitated, by the vast amounts of data that are readily available from processes, increased digitalization, automation, increasing computation power, and simulation software that can model complex phenomena that span over several temporal and spatial scales. Although this paper is not a comprehensive review, it gives an overview of the recent history of machine learning models that we use every day and how they shaped process design problems from the recent advances to the exploration of their prospects.

## A BRIEF HISTORY OF MACHINE LEARNING

The roots of machine learning (ML) can be traced back to the early 19^th^ century when the method of least squares was first discovered by Legendre in 1805 and later by Gauss in 1809 [1]. However, the main concept of computers learning from experience without explicitly being programmed has roots tracing back to more recent history, the mid-20^th^ century.

The foundational idea of neural networks emerged in the 1940s and 1950s when researchers began exploring mathematical models inspired by the structure and functioning of the human brain. Warren McCulloch and Walter Pitts' paper, "A Logical Calculus of Ideas Immanent in Nervous Activity," was published in 1943, where they proposed a mathematical model of an artificial neuron which was the first idea of using a computational model for neural networks [2]. This foundational paper laid the groundwork for subsequent developments in neural network theory. The term "neural network" itself gained prominence in the 1950s and 1960s as researchers like Frank Rosenblatt developed the perceptron, an early form of a neural network designed for pattern recognition tasks. While the perceptron had limitations, the idea of using computational models to simulate neural processes became a cornerstone in the evolution of artificial neural networks (ANNs) and ML. Around the same timeline, response surface methodology was introduced by Box and Wilson [3], and the term “machine learning” was coined by Arthur Samuel [4].

Throughout the following decades, various modeling approaches and algorithms, including Gaussian process (GP) regression, backpropagation algorithm, support vector machines (SVMs), and Random Forest (RF), emerged in the ML landscape. Especially, the establishment of the backpropagation algorithm was a pivotal moment in the resurgence and widespread adoption of neural works starting 1980s, enabling researchers to revisit the complex problems that were not possible to address before. This ultimately led to the application of neural networks in various scientific and engineering domains, including process design and operations, and to the development of more complex algorithms. These include reinforcement learning, deep learning, natural language processing, and generative artificial intelligence (AI) which are emerging areas of research within process systems engineering [5,6]. Although NN models have been the primary modeling strategy in process design problems due to their ability to capture nonlinearities very accurately, we will also demonstrate that SVMs and tree-based ensemble models like RF and gradient boosted trees are also studied in depth.

## PAST APPLICATIONS OF ML IN PROCESS DESIGN

With these developments underway, it was also imperative to revisit optimal design problems from the lens of ML models and process synthesis. Regression analysis and parameter estimation for kinetic or thermodynamic models were already performed for process design, dating back to the 1960s. However, with the increasing computation power and the development of process simulation software, optimal design problems recognized the need for surrogate ML models due to: (1) the “black-box” nature of the simulation software that lacks the derivative information that is imperative for optimization; (2) the computational expense associated with sample-based derivative-free optimization techniques; and (3) the high mathematical complexity of process synthesis problems (mixed-integer nonlinear program – MINLP) that become intractable with high number of variables and constraints. Hence, earlier introduction of ML techniques in process design focused on replacing highly complex and/or noisy simulations with relatively simpler representations, especially within optimization frameworks to alleviate the mathematical complexity.

For example, Caballero and Grossmann used kriging surrogate models to replace noisy unit operations in modular flowsheet optimization [7]. Likewise, Davis and Ierapetritou used kriging surrogates for tertbutyl methacrylate production design and process synthesis [8]. Henao and Maravelias used ANN surrogate models trained using the data collected from the process simulator to replace complex unit operations (*e.g*., distillation column, expansion valves, heaters/coolers, flash vessels, absorption columns) and reformulated these ANNs to incorporate within their superstructure optimization framework [9]. Fahmi and Cremaschi also used ANNs to substitute for thermodynamics and mixing models, as well as unit operations for process synthesis of biodiesel production [10]. One of the key challenges using ANNs was also noted in this work, where these models were “data hungry” (*i.e*., large amounts of data were required to train accurate ANN representations). The modeling complexity of the ANNs also made it challenging to incorporate them in large-scale optimization problems without any efficient reformulation strategies. Equation 1 shows the mathematical structure of a general feed-forward NN, represented by a repeated composition of functions,

(1) $$y=f(x;\theta )=f_{L}\circ f_{L-1}\circ \ldots \circ f_{2}\circ f_{1}(x;\theta )$$

where y is the output of the network, x are the inputs to the network, $f_{i}$ is a layer in the neural network with transformations applied by the activation functions, and θ are the weights and biases for the entire network. In simpler terms, this mathematical structure generates highly nonlinear expressions (except for purely linear activation functions) that create additional complexities for optimization algorithms to handle (Equation 2).


(2) $$y=f_{L}\left(f_{L-1}\left(f_{L-2}\left(\ldots .f_{1}(x)\right)\right)\right)$$

Especially, within a global optimization framework, this nested functional form can be intractable as well. Motivated by this, most recent progress focused on using more simplified surrogate models for process design and synthesis problems, as well as developing novel reformulation strategies that exploit the mathematical properties of activation functions. Next, we discuss these developments and other key progress in this area.

## CURRENT PROGRESS

### Reformulation of ML Models

One of the most recent key breakthroughs in using ML in any optimization framework (*e.g*., process design, synthesis, or operations) is the ability to reformulate deep NNs with rectified linear unit (ReLU) activation functions into a mixed-integer linear program (MILP) [11–13]. By recognizing ReLU activation functions as max-affine spline operators that are piecewise linear (Equation 3), ANNs can be exactly reformulated with big-M constraints to create a MILP that can be solved to global optimality with off-the-shelf solvers.


(3) $$y=max\{0,z\}=\left\{\begin{array}{ll} 0 & if\;z<0\\ z & otherwise \end{array}\right.$$

**Figure 1.**
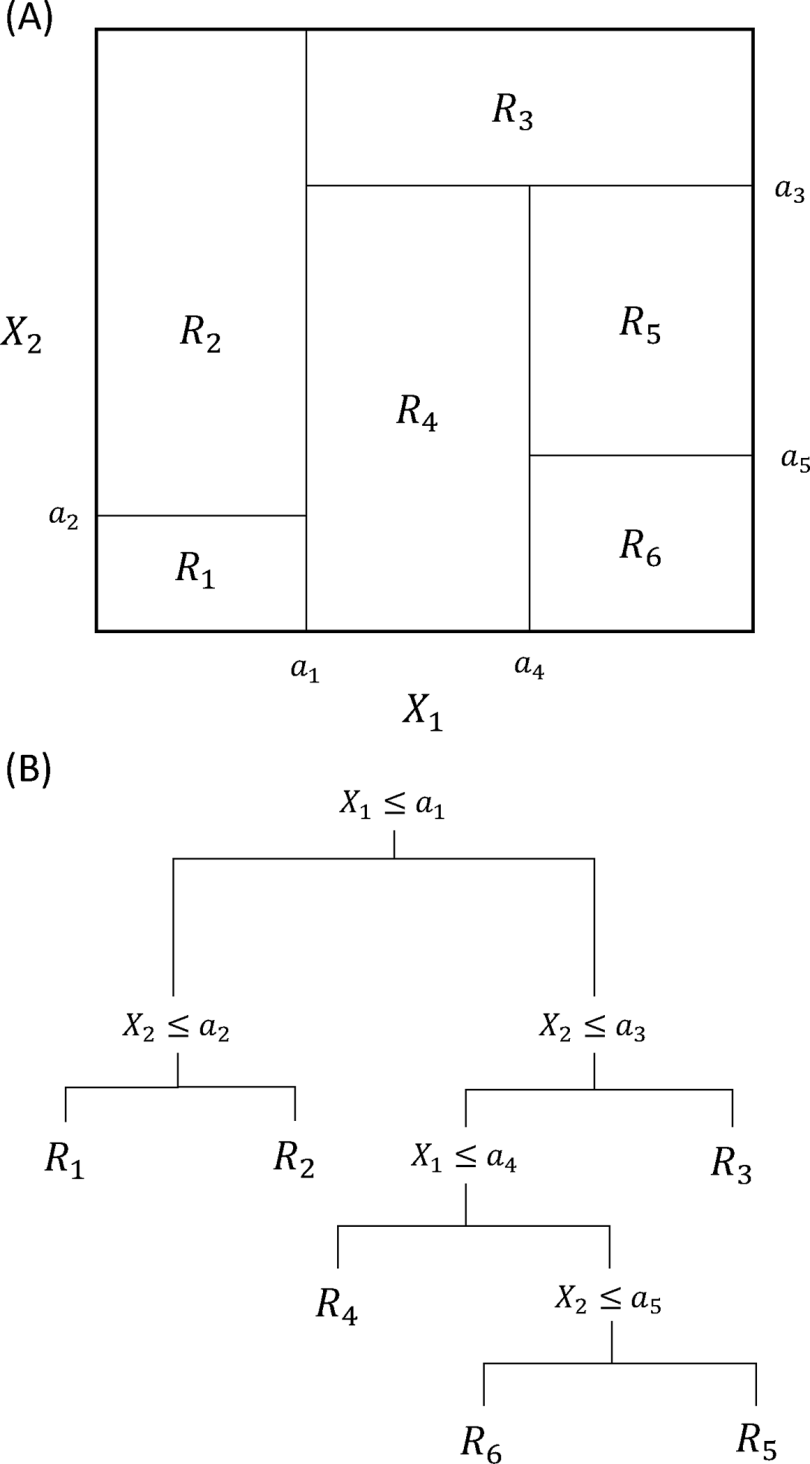
A demonstration of how decision tree models (A) partition the space with piecewise linear models; and (B) map this partitioning onto decision trees for a visual representation.

This enabled MILP-reformulated ANNs to be embedded in a variety of problems, including optimizing the extractive distillation process [14], sustainable hydrogen production using sorption enhanced steam methane reforming [15], as well as for modeling flexibility index constraints in biorefinery design by superstructure optimization [16]. This technique is also extended to other activation functions that are nonlinear [17,18], and different modeling strategies such as tree-based ML models, as they also partition the modeling space with piecewise linear models (Figure 1).

Mišić [19] and Mistry *et al*. [20] encoded trained gradient boosted regression trees, which are ensemble decision tree models), to MILP models that are later embedded into optimization problems. This technique is further extended as a black-box optimization algorithm in the ENTMOOT framework [21] and implemented as an opensource software package named OMLT [22]. The applicability of the tree-based reformulation is also demonstrated on an optimal layout design problem of an offshore windfarm [23].

Despite reformulation strategies alleviating a portion of the nonlinearity issues in NN and tree-based ensemble models, we also observe that large-scale process synthesis problems still rely on more simplistic models. For instance, Demirhan *et al*. used linear surrogate models to model the conversion of a Haber-Bosch reactor within the renewable ammonia process synthesis problem that has 18,573 continuous, 38 binary variables, and 18,924 constraints [24]. Under such large-scale global optimization problems, reformulating MILP representations of NN or tree-based models of individual units will amplify the number of binary variables, which will further increase the complexity of the overall optimization model. Hence, the use of ML in large-scale synthesis problems is still contingent on the overall problem complexity, even when ML models offer highly accurate predictions.

### ML Algorithms as Constraints

Nowadays, the use of ML is not limited to modeling individual unit operations or an entire flowsheet, but it can also serve as constraints to process design problems. Especially, process simulations are typically subject to black-box constraints that lack explicit analytical expressions relating the decision variables to constraint violations (*i.e*., the constraint violations can only be obtained once the simulation run is completed). Constraint handling can be achieved in many ways, including augmented Lagrangian formulations, penalty, filter, or barrier methods [25].

**Figure 2.**
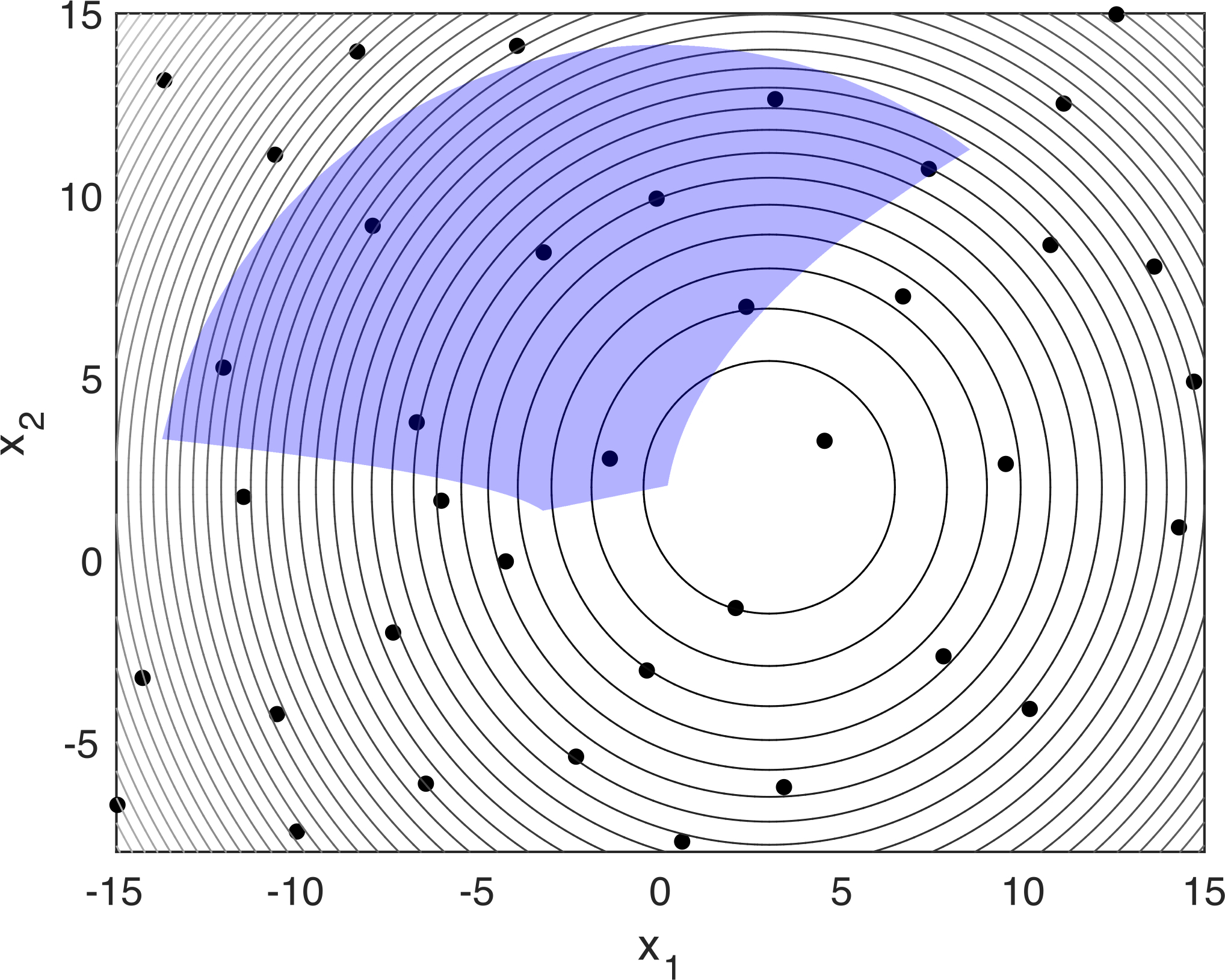
The feasible region derived by fitted surrogates using regression analysis (purple) is shown on a contour plot of the objective function. Black dots show the sampling points for the input space. The constraints are: $x^{2}+y^{2}-200\leq 0$; x−5y+10≤0; $25x-2y^{2}+4y-5\leq 0$; $-x-2y^{2}+4y-5\leq 0$.

**Figure 3.**
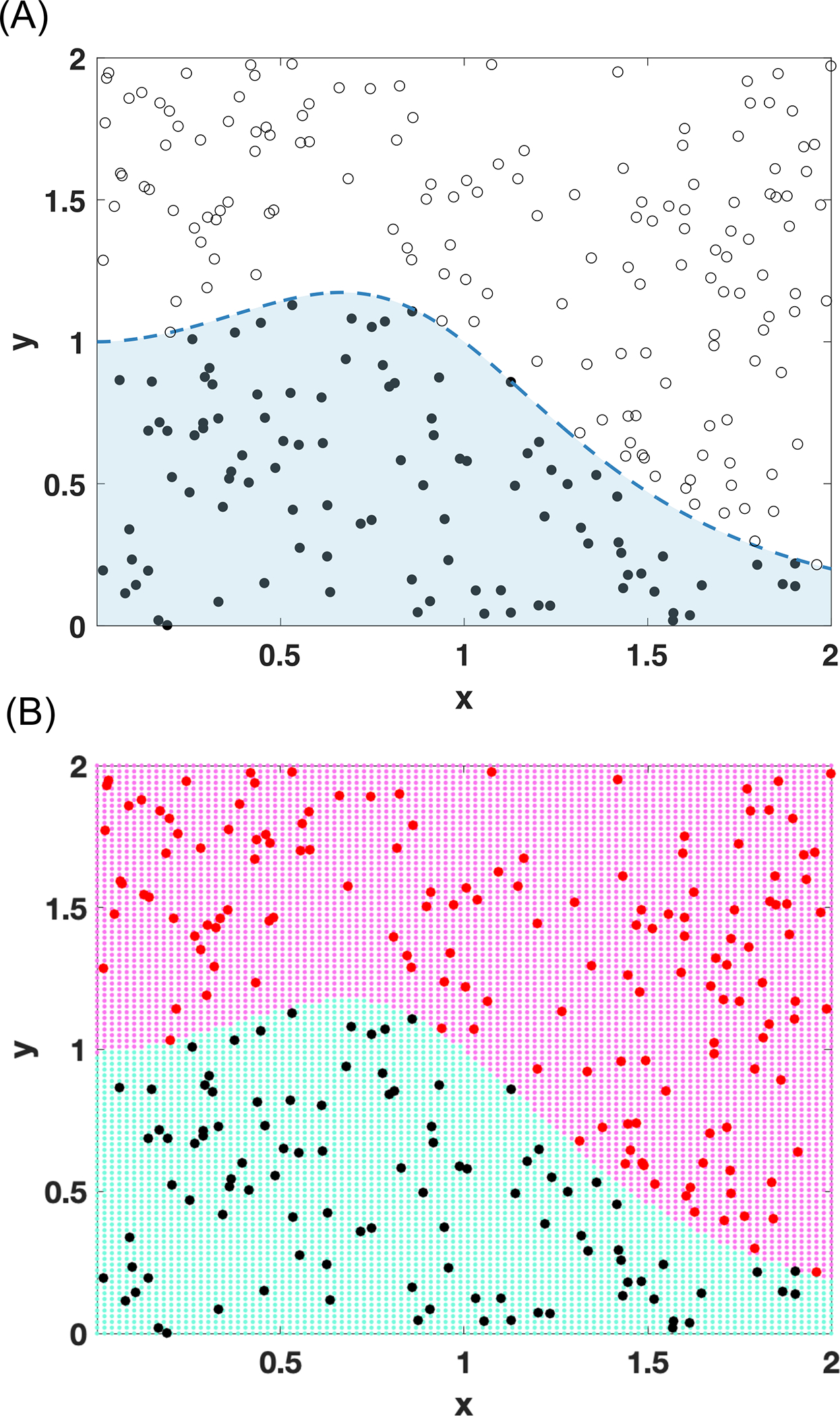
An SVM-based classifier trained to mimic the nonlinear constraint, $y<1/\left(x^{3}-x^{2}+1\right)$: (A) The original constraint within the bounded space; (B) The map of the feasible region captured by SVM. The predictive performance of the classifier on a blind testing set: Accuracy = 100%; Sensitivity = 100%; Specificity = 100%; F_1_ score = 100%.

On the other hand, ML tools can be leveraged to handle constraints individually as a regression task [26,27], where constraint violations are modeled as less than or equal to constraints with surrogate models (Figure 2), or holistically as a classification task [28–30], where a separating model between feasible and infeasible solutions are established. A conceptual demonstration of a nonlinear constraint being modeled as a classifier using SVM with Gaussian radial basis function kernel is provided in Figure 3.

This is achieved by using a dataset of simulated samples with their binary outcome (feasible/infeasible) to train one or more classification models instead of individually modeling actual violation values as done in the regression analysis. The trained classifier can then be used with a data-driven optimizer or implemented in a superstructure model using the aforementioned reformulation strategies. In that respect, SVM classifiers are shown to effectively model the implicit constraints of numerically infeasible differential algebraic equations of a steam cracker reactor design problem [28,29]. SVM classifiers are also used for modeling feasibility constraints in the vertex formulation of modular design problems [30].

While these studies show promise for using classifiers, the offline model training is still time-consuming (*i.e*., several thousand samples are collected from the simulator to create the model) with no efficient way to recycle or integrate the already collected data into the decision-making process. Also, understanding the uncertainties surrounding these models as well as their misclassification rate is of utmost importance for constraint modeling, as misclassifications can lead to infeasible solutions or designs, whereas in regression-based models, such misviolations are less likely to happen.

### Large Language Models & Generative AI for Process Design

Choosing the right sequence of unit operations and connections to create a flowsheet is a fundamental practice in process design, whether it is done heuristically or through superstructure optimization. With the launch of ChatGPT, the key question becomes whether natural language processing or generative techniques can be utilized for process design and discovery. As a language model, ChatGPT is designed to understand and generate human-like text based on the input it receives. However, chemical engineering problems, such as process flowsheet generation, are based on recognizing the sequence of unit operations. This comes with the caveat of lack of suitable data to be able to train the large language models that can generate a flowsheet automatically [31]. For instance, there could be 20+ different representations for the same unit [32] or process flowsheets are most likely proprietary or unavailable to extract the information necessary for the AI model development [33].

To overcome these limitations, Vogel *et al*. developed SFILES 2.0 [33] to represent flowsheets using a graph notation, analogically similar to the text-based SMILES notation for representing chemical structures. This was primarily developed to topologically describe a flowsheet with the disadvantage of not storing any information about the sizing or the operating conditions of the units. Along the idea of ChatGPT, Vogel *et al*. also investigated the automatic completion of flowsheets using causal language modeling [34]. Their results show that the generative AI model can learn the topological patterns in flowsheet data and can automatically complete flowsheets. However, like the issues faced in ChatGPT, the generated flowsheet may not make practical sense, as the developed model does not intake contextual information about the process. Using the SFILES notation, Hirtreiter *et al*. also investigated the automatic generation of control structures for flowsheets [35]. While the predictive accuracy of the trained models was relatively high, significant limitations are also noted by the authors. Especially, concerns regarding safety indicators, understanding the process dynamics and operational objectives, and lack of information on the equipment sizing and operating conditions for the units pose major questions. These promising developments show that natural language processing and large language models can provide a “warm start” for flowsheet generation and facilitate some of the time-consuming tasks. However, more research needs to be done in this area to be able to improve confidence in model predictions while providing a holistic view of process design beyond just the topological investigation.

## CONCLUSIONS

Artificial Intelligence and machine learning (ML) models are now an essential component of process design with efficient model integration and reformulation strategies paving the way. Constraint handling with ML models to flowsheet generation using large language models, we see new and innovative ways of how ML is used for process design problems. While the main motivation for using ML models is to alleviate the model complexities and such models have proven to be successful over the course of decades, domain knowledge and model interpretability will still play the most important role despite the promise these models hold. The ability to understand why a model makes a particular prediction and to reason if predicted results are physically sound or whether a generative model-derived process flow diagram is safe to implement becomes critically important. This judgment requires a deep fundamental understanding of the process, engineering expertise, and other relevant domain knowledge. Incorporating safety and risk measures, and combining ML models with first-principles information to create hybrid models are a few avenues that researchers are currently investigating. Nevertheless, the importance of interpretability and understanding the process relevance of the predictions will persist in this field.
